# Reassessment of the Lineage Fusion Hypothesis for the Origin of Double Membrane Bacteria

**DOI:** 10.1371/journal.pone.0023774

**Published:** 2011-08-18

**Authors:** Kristen S. Swithers, Gregory P. Fournier, Anna G. Green, J. Peter Gogarten, Pascal Lapierre

**Affiliations:** 1 Department of Molecular and Cell Biology, University of Connecticut, Storrs, Connecticut, United States of America; 2 University of Connecticut Biotechnology Center, University of Connecticut, Storrs, Connecticut, United States of America; 3 Department of Biological Engineering, Massachusetts Institute of Technology, Cambridge, Massachusetts, United States of America; J. Craig Venter Institute, United States of America

## Abstract

In 2009, James Lake introduced a new hypothesis in which reticulate phylogeny reconstruction is used to elucidate the origin of Gram-negative bacteria (Nature 460: 967–971). The presented data supported the Gram-negative bacteria originating from an ancient endosymbiosis between the Actinobacteria and Clostridia. His conclusion was based on a presence-absence analysis of protein families that divided all prokaryotes into five groups: Actinobacteria, Double Membrane bacteria (DM), Clostridia, Archaea and Bacilli. Of these five groups, the DM are by far the largest and most diverse group compared to the other groupings. While the fusion hypothesis for the origin of double membrane bacteria is enticing, we show that the signal supporting an ancient symbiosis is lost when the DM group is broken down into smaller subgroups. We conclude that the signal detected in James Lake's analysis in part results from a systematic artifact due to group size and diversity combined with low levels of horizontal gene transfer.

## Introduction

Symbioses and endosymbioses have shaped and continue to shape microbial evolution [1]. As such, it is of little surprise that endosymbiotic events and chimaerism are often considered useful hypotheses for explaining the phylogenetic and gene content complexities of bacterial, archaeal and eukaryotic genomes. James Lake used a reconstruction of reticulate phylogeny to argue that the double membrane bacteria evolved from an ancient symbiosis (endosymbiosis) between Clostridia and Actinobacteria [2]. By applying a parsimony analysis of protein family presence absence data over five distinct groups of prokaryotes [3], he identified sets of proteins present in double membrane bacteria (DM) that originated from either Clostridia or Actinobacteria. Since the highest number of protein families from the presence-absence patterns had better support for a ring structure compared to a single bifurcating tree, he concluded that the most likely explanation for the data was a fusion event between Clostridia and Actinobacteria. If this fusion occurred through an endosymbiosis, it could also explain the origin of the double membrane architecture. This view has been supported by interpreting polarizing indels (insertions or deletions) within several protein families as excluding the bacterial root from within Actinobacteria and DM bacteria [2], [4], compatible with a monophyletic fusion origin of DM bacteria with Actinobacteria as a participating lineage. Additionally, it was argued that the photosynthetic machinery would resist being transferred because of its complexity, and thus be a good candidate to study ancient divergences [2]. These assumptions and the results of the aforementioned analyses have not gone without criticism, on both theoretical and methodological grounds [5].

One problem with this analysis is that the group designated DM is comprised of many rather divergent groups of bacteria, such as the Dictyoglomi, Thermotogae, *Deinococcus-Thermus*, Cyanobacteria and the different classes of Proteobacteria (see materials and methods for full listing). The definition of what constitutes a genuine double membrane compared to an external proteolipid or protein layer is unclear, and the constituents of the outer layer are difficult to determine [6]. For this reason, the majority of phyla included as double-membrane organisms are controversial and have possibly introduced an artifactual signal in favor of a fusion. Given the amount of interdomain and interphylum horizontal gene transfer that has been identified (e.g., [7], [8], [9]), one should expect a larger group of organisms to harbor more different protein families than a smaller group. This alternative explanation for Lake's data is testable; if the reticulate signal detected by Lake were due to many transfers of individual genes and operons, it should diminish if the DM group is replaced in the analysis with any of its biologically cohesive constituent subgroups. In contrast, if the signal were due to a single ancient endosymbiotic event at the root of the DM bacteria, then the signal should not disappear even if only a subgroup of the DM were selected in the analysis.

**Figure 1:**
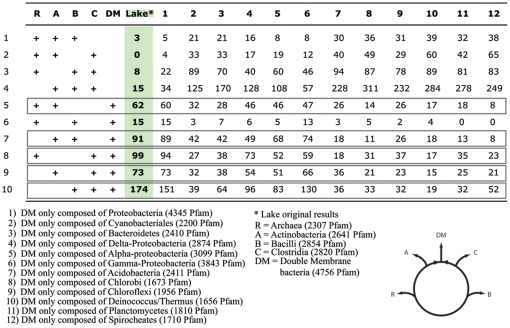
Protein family counts for the ten possible informative profiles. The table was adapted from Lake's Table 1 [2] to include the Pfam counts that result if different representative classes are chosen for the DM group. Number of Pfam per group is in parentheses the same number as in Lake's paper was found for all other groups. The circle illustrates Lake's hypothesis that the double membrane bacteria resulted from a fusion between Clostridia and Actinobacteria. The patterns compatible with this hypothesis are boxed (pattern 5,7,8,9 and 10).

The claim that DM bacteria evolved from an ancient symbiosis is based on an analysis that aggregates all Bacteria and Archaea into 5 groups (the double membrane prokaryotes (DM), Actinobacteria (A), Bacilli (B), Clostridia (C), Archaea (R)), using the Pfam database [10] to determine the number of protein families that were represented in 3 out of the 5 aggregate groups. A protein family (Pfam) was considered present in a group, if at least one genome within the group encoded a member of this family. The analysis produces a table of all possible combinations of presence-absence profiles and determines the most parsimonious scenario explaining the data (i.e., if they were generated by a tree-like or ring-like evolutionary process, see supplemental Figure S1). The ring structure proposed by Lake [2] joins the DM group to both the A and C groups given the allowed patterns in rows 5, 7, 8, 9 and 10 (Figure 1). The presence of a higher number of genes in those five rows compared to the tree signal (rows 1, 4, 7, 8, 9 and 10 in Lake 2009, Figure S2) reflects a higher number of genes shared by DM members with Actinobacteria and Clostridia. If the argument for a fusion event were valid, trends observed in the gene presence-absence table should not be affected by the breakup of the DM into sub-groups, as the presence of the protein family would be shared derived characters of all DM members.

## Results

**Figure 2:**
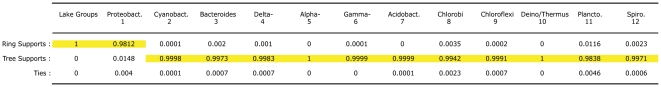
Posterior bootstrap support values (p-values) for a ring model, tree model or equal probabilities for each of the sampled groups. The p-values were calculated from 10,000 re-samplings with replacement and extracting the total number of times the tree model, ring model or when both were equally supported from the parsimony counts. Only in the case where all the double membrane prokaryotes as defined by Lake [2], or when all the proteobacteria were included, did a ring model better explain the data.

We repeated Lake's analysis exactly using the same version of the Pfam database, and in addition to Lake's DM group, we also analyzed the datasets that resulted after dividing the DM group into twelve subclasses (Figure 1, column one to twelve). We found that for most of the DM subgroups, tree patterns were more highly supported than the patterns allowed under the ring scheme proposed by Lake. Additionally, the signal supporting the hypothesis of an ancient endosymbiosis between Clostridia and Actinobacteria is completely lost (p-values in favor of a ring of 0.0035 or smaller) when these subgroups are used as representatives for the DM group (Figure 2 and S2). This result is compatible with the hypothesis that the reticulate signal is due to several HGTs of individual genes, operons, and gene clusters and not due to a single ancient fusion between lineages. The ring signal is retained only in one case, when all classes of the Proteobacteria are combined (p-value of 0.98), possibly because this group contains the largest sampled biodiversity as reflected by the number of protein families in Pfam compared to the other groups included in the analysis. Figure 2 summarizes our results. We conclude that the deduced reticulate phylogeny appears to be due to many individual gene transfer events. The division of prokaryotes into groups of different size and containing different amounts of sampled protein diversity produces a systematic artifact suggestive of a fusion at the base the group comprised of the most diverse members.

## Discussion

Lake's result that the DM group arose via a fusion event implies that this group be monophyletic rather than paraphyletic, since this model is inconsistent with the DM ancestor giving rise to any other groups of bacteria included in the analysis. However, while it has been claimed that a polarizing indel within the HSP70/MreB gene families excludes the root of the “tree of life” from gram-negatives [11] it is likely that this result is impacted by extensive horizontal gene transfer, and is complicated by alignment and sampling artifacts [12], [13]. More convincingly, a polarizing indel in the HisA/HisF protein families and the quaternary structure of PyrD homologs have also been used to exclude the root from most gram-negatives and actinobacteria [14]. While the results of these analyses do permit the monophyly of the DM group, they also permit any scenario where each DM subclass is derived, including a paraphyly or even polyphyly incompatible with the assumptions in [2]. For these reasons, indels do not provide support for a monophyletic DM group as described in (Lake 2009). The argument that the photosynthetic machinery is reluctant to gene transfers because of its complexity, thus linking the Clostridia from one side of the ring of life to the DM bacteria, is similarly weak: previous reports have shown that many photosynthesis genes, including the chlorophyll biosynthetic pathway were transferred between bacterial classes and phyla [15], [16]. Sharon *et al*. have even reported the discovery of a complete photosystem I operon in a marine phage [17], and analysis by Igarashi *et al*. [18] suggested that a photosynthetic gene super-cluster in the β-Proteobacteria was acquired through transfer from the α-Proteobacteria.

Endosymbiosis between two single-membrane organisms as proposed by Lake [2] offers a possible scenario for the evolution of double membranes as a derived character possessed by descendants of the fusion event. Our analysis using subclasses of DM bacteria shows that the parsimony approach of Lake [2] only found an origin by fusion for groups of organisms containing a large amount of protein diversity, as evidenced by our recovery of a predicted fusion event only for the Proteobacteria subclass, and not for any other constituent subclass of DM bacteria. Therefore, presence-absence analysis of protein families does not provide evidence of reticulate evolution between Actinobacteria and Clostridia.

## Materials and Methods

The complete Pfam database v.22.0 was downloaded from ftp.sanger.ac.uk/pub/databases/Pfam/releases/Pfam22.0/and was locally searched for presence of protein families across different groups. We divided the Pfam database into five groups according to Lakes specifications as described in [2]. Group 1 was composed of all the Archaeal protein families; group 2 was composed of the Actinobacteria; Group 3 are the Bacilli which includes the Lactobacillales and the Bacillales; Group 4 was represented by the Clostridia and Mollicutes which also included the *Symbiobacterium*, Coriobacteriales and the Rubrobacteridae; and finally group five which represents all the double membrane prokaryotes (Acidobacteria, Aquificae, Bacteroidetes, Chrysiogenetes, Chloroflexi, Chlorobi, Chlamydiae, Cyanobacteria, Deferribacteres, Deinococcus/Thermus, Dictyoglomi, Fibrobacteres, Fusobacteria, Nitrospirae, Proteobacteria, Planctomycetes, Spirochaetes, Thermodesulfobacteria, Thermotogales and Verrucomicrobia). Figure 1 shows the ten parsimonious informative character states for the five group comparisons. Following Lake's methods, a protein family was deemed present if at least one member of the three subject groups contained that protein family and was absent in all members of both query groups. Using the original group classification, we recovered the exact numbers of protein families for the ten-character state as described by Lake. We then compiled the number of protein families present when the double membrane group was broken up into twelve subgroups: Proteobacteria, Cyanobacteria, Bacteroidetes, δ-Proteobacteria, α-Proteobacteria, γ-Proteobacteria, Acidobacteria, Chlorobi, Chloroflexi, Deinococcus/Thermus, Planctomycetes and Spirocheates. The posterior bootstrap support values (p-values) for all possible ring and tree models were calculated from 10,000 re-samplings with replacement and extracting the total number of times the tree model, ring model or both were equally supported. For each bootstrap replicate, the best supported model was determined by finding the tree or ring with the lowest minimum parsimony count. The minimum parsimony counts were calculated by weighting the number of Pfams supporting a particular tree or ring twice that of the number of Pfams that do not support the model [3].

## Supporting Information

Figure S1 List of all possible trees and rings for five taxa sampling. Each possible tree and ring is listed with the compatible presence-absence pattern of gene families (Pfam) given in Figure 1. For example, the tree and ring corresponding to ABCDR are shown at the left of each table. A corresponds to Actinobacteria, B to Bacilli, C to Clostridia, D for double membrane prokaryotes and R for Archaea.
(TIF)

Figure S2 Minimum parsimony counts supporting each of the possible trees (A) and rings (B). The lowest count is used to determine if the data supports a tree or a ring [3]. In the original analyses by Lake [2], the best ring had a minimum parsimony count of 581 versus 625 for the best supported tree (first column). Best supported trees or rings for each tested case are highlighted.
(TIF)
