# Supplementary figures and images for: Reassessment of the Lineage Fusion Hypothesis for the Origin of Double Membrane Bacteria
Source: PLoS One. 2011 Aug 18;6(8):e23774. doi: 10.1371/journal.pone.0023774 (PMC3158100; doi:10.1371/journal.pone.0023774)

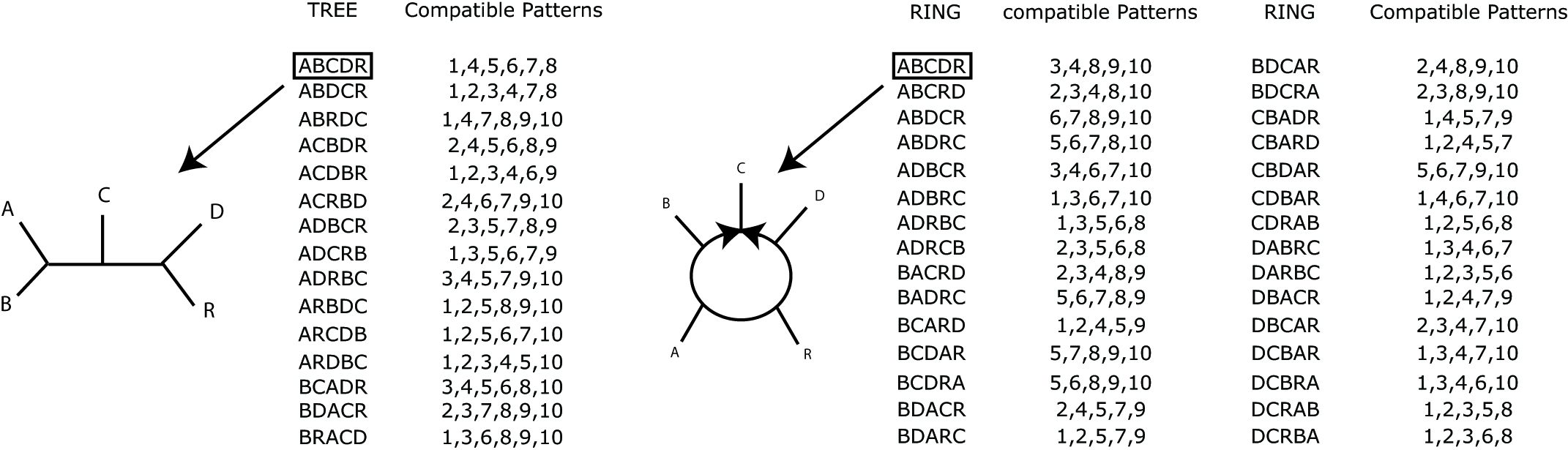

Supplement: Figure S1 — List of all possible trees and rings for five taxa sampling. Each possible tree and ring is listed with the compatible presence-absence pattern of gene families (Pfam) given in Figure 1. For example, the tree and ring corresponding to ABCDR are shown at the left of each table. A corresponds to Actinobacteria, B to Bacilli, C to Clostridia, D for double membrane prokaryotes and R for Archaea. (TIF) [file pone.0023774.s001.tif]

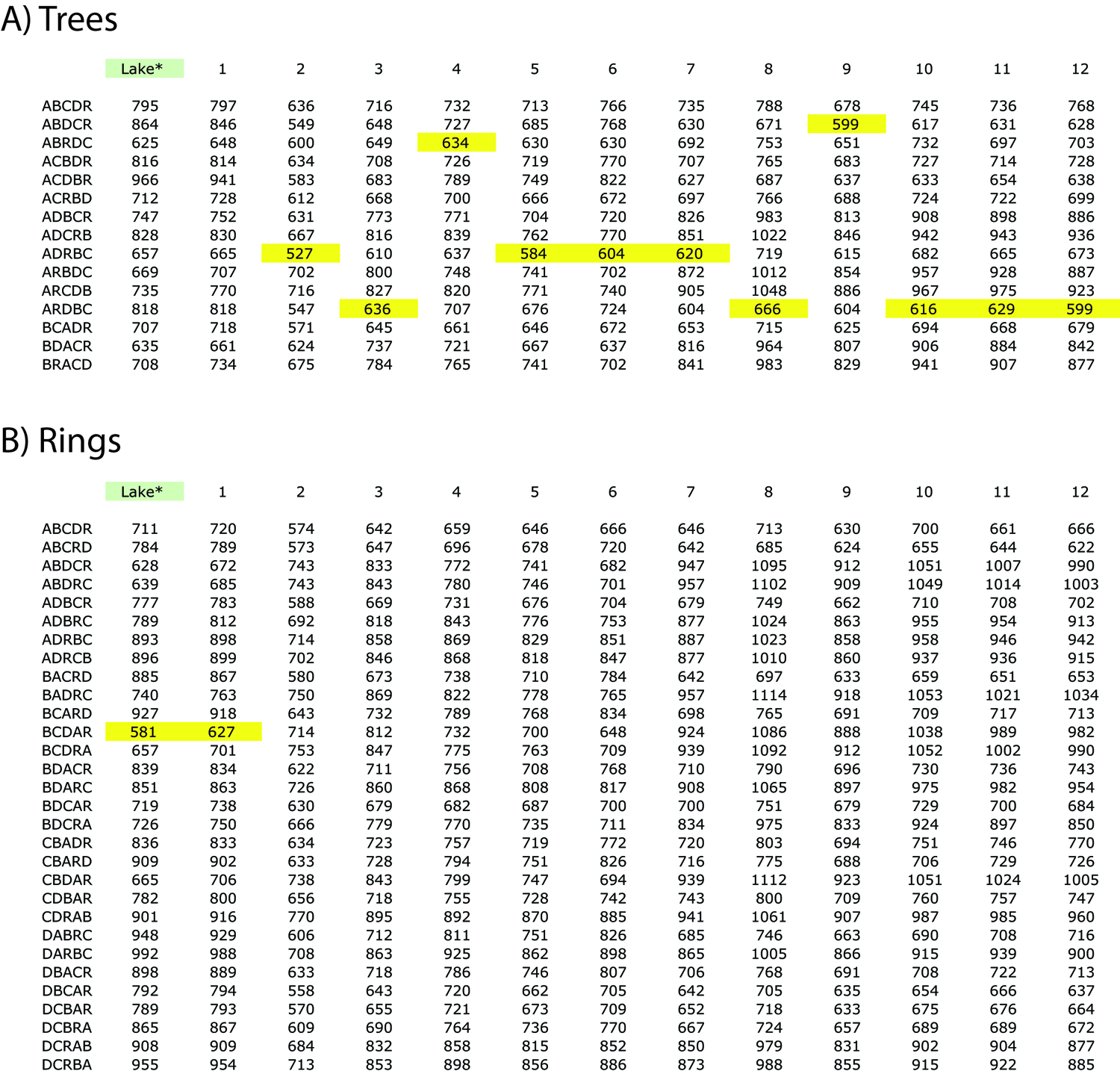

Supplement: Figure S2 — Minimum parsimony counts supporting each of the possible trees (A) and rings (B). The lowest count is used to determine if the data supports a tree or a ring [3]. In the original analyses by Lake [2], the best ring had a minimum parsimony count of 581 versus 625 for the best supported tree (first column). Best supported trees or rings for each tested case are highlighted. (TIF) [file pone.0023774.s002.tif]
